# The clinico-pathologic profile of primary and recurrent orbital/periorbital plexiform neurofibromas (OPPN)

**DOI:** 10.1371/journal.pone.0258802

**Published:** 2021-10-21

**Authors:** Mohammad Alabduljabbar, Diego Strianese, Osama Al-Sheikh, Hind M. Alkatan, Hailah Al-Hussain, Azza M. Y. Maktabi, Rajiv Khandekar, Malak Abedalthagafi, Deepak P. Edward

**Affiliations:** 1 King Khaled Eye Specialist Hospital, Riyadh, Saudi Arabia; 2 King Saud University, College of Medicine, Riyadh, Saudi Arabia; 3 Genomics Research Department, Saudi Human Genome Project, King Fahad Medical City and King Abdulaziz City for Science and Technology, Riyadh, Saudi Arabia; 4 Department of Ophthalmology, Visual Sciences and Pathology, University of Illinois, College of Medicine, Chicago, IL, United States of America; BIDMC, UNITED STATES

## Abstract

To evaluate and compare the clinical and histopathological profile of primary and recurrent orbital-periorbital plexiform neurofibromas (OPPN) in patients with neurofibromatosis type 1. We retrospectively evaluated 43 primary or recurrent neurofibroma (NF) specimens from 26 patients (2002 to 2018) at the King Khaled Eye Specialist Hospital, Saudi Arabia. Demographics, clinical presentation, and surgical intervention data were collected. Histopathological specimens were studied with hematoxylin-eosin, Alcian blue, and immunohistochemical markers; S-100, CD44, CD117, smooth muscle actin (SMA), neurofilament, and Ki-67. Of the 43 NFs specimens, 20 were primary and 23 recurrent tumors. For primary NF, the ratio of plexiform to the diffuse type was 13:7, however in recurrent tumors was 3:8 after the first recurrence, and 1:5 after multiple recurrences. Of the 17 patients with primary tumors that had paired recurrent tumors, 12/17 (70.6%) primary NFs were plexiform and 5/17 (29.4%) were diffuse. However, when tumors recurred, 13/17 tumors (76.5%) were diffuse and only 4/17 tumors (23.5%) had a plexiform pattern. The odds of a tumor having a diffuse pattern in recurrent NF was significantly higher than the plexiform pattern [OR = 7.8 (95% confidence interval 1.69:36.1) *P = 0*.*008*]. Primary plexiform NFs underwent an excision at a significantly younger age than the diffuse type. Recurrent NFs had significantly higher CD44, CD117, and neurofilament labeling (*P = 0*.*02*, *P = 0*.*01* and *P<0*.*001* respectively) but had significantly decreased Alcian blue, and S-100 labeling (*P = 0*.*03*, and *P = 0*.*02* respectively) compared to primary tumors. SMA and Ki-67 proliferation index were not different between primary and recurrent NFs (*P = 0*.*86*, and *P = 0*.*3* respectively). There appears to be a high risk for primary plexiform NFs to develop a diffuse histologic pattern when they recur. Immunohistochemical staining suggests a role of mast cells (CD117) and expression of infiltration makers (CD44) in the transformation of plexiform tumors to the diffuse phenotype.

## Introduction

Neurofibromatosis type 1 (NF1; von Recklinghausen disease) is an autosomal dominant disorder that affects 1 in 3500 births worldwide [1–3]. Orbital-periorbital plexiform neurofibromas (OPPN) affects about 10% (1–22%) of patients with NF1 and can cause devastating functional and cosmetic effects secondary to ptosis, proptosis and facial disfigurement leading to social and emotional distress to patients and their families [4,5]. OPPN is classified based on gross and microscopic appearance as plexiform, diffuse and localized types of neurofibromas (NFs) [5–8].

Plexiform neurofibromas (PNs) are irregular, ill-defined, slowly growing lesions that often involves periorbital tissues. Histologically, these tumors are composed of complex intertwining bundles of enlarged nerves with a proliferation of Schwann cells and endoneurial fibroblasts in a mucoid matrix surrounded by perineurium [8–10]. Diffuse NFs are poorly defined, infiltrating tumors that expand along connective tissue septa and intercellular spaces, replacing orbital fat and permeating extraocular muscles without destroying the tissue. This tumor has a vascular component and carries minimal risk of malignant transformation. Histologically, it is similar to plexiform in its cellular components but differs in that it has a uniform matrix of fine fibrillary collagen and sometimes contains pseudo-Meissner corpuscles [8–10]. Localized NFs are well-circumscribed, non-encapsulated, lesions that is not associated with NF1. It is rare in the orbit and has minimal risk of malignant transformation [11,12]. Little is understood about the factors involved in the recurrence of NFs. Genetic and environmental factors are thought to induce hyperproliferation of Schwann cells in NFs [13,14].

Neurofibroma tends to recur with increasing risk in patients younger than 10 years of age and in partially resected tumors especially the diffuse ones. The rate of recurrence is 15–23% in general and 43% in children [15–17]. Usually NF is worse when it recurs however, the changes behind these recurrences are poorly understood.

We present the clinical and histopathological features of primary and recurrent OPPNs and used the information to understand the clinical and cellular behavior of recurrent tumors.

## Materials and methods

We retrospectively reviewed all patients diagnosed with NF1 who underwent surgical debulking for NFs as primary or recurrent procedures from 2002 to 2018 at King Khaled Eye Specialist Hospital (KKESH), a tertiary eye care referral center in Riyadh, Saudi Arabia. NF1 diagnosis was made using criteria established by the National Institute of Health [18]. The study was approved (Project number 1809-R) by the Human Ethics Committee/Institutional Review Board at KKESH, Riyadh, Saudi Arabia. The inclusion criteria were patients diagnosed with NF1 who underwent surgical debulking of histologically diagnosed NF either primary or recurrent from 2002 to 2018 at KKESH. The exclusion criteria were patients who did not have the classic histological pattern of NF.

For this study, we identified patients meeting the inclusion criteria from KKESH Pathology Department archives. We retrieved the medical records and reviewed the clinical data, including demographics, ophthalmologic examination, and surgical interventions. We then classified tumors into primary and recurrent tumors. In patients who had the primary tumor removed before 2008, tissue blocks were unavailable since they were disposed according to the hospital policy, we used those clinical details from records and pathology reports for these cases to define the type of NF.

Hematoxylin and eosin (H&E) stained slides were examined under the light microscope to identify the pattern of NFs (plexiform or diffuse). We also classified tumors that had both features of plexiform and diffuse as mixed NFs. We also looked at primary tumors and paired them with recurrent tumors from the same patient for analysis where tissue/slides were available for review by the ophthalmic pathologist and performed additional analysis to study the relationship between the conversion of plexiform tumors to diffuse tumors.

### Histochemical stains and immunohistochemistry

Histochemical stains were performed in the pathology department at KKESH and immunohistochemical (IHC) staining were performed in the genomics department at King Fahad Medical City (KFMC). Alcian blue was used to identify and compare the myxoid component of primary and recurrent NFs [19–21]. IHC studies and the rationale for use in this study are listed in Appendix 1.

S-100, CD44, and Ki-67 slides were reviewed and scored by two pathologists (DE AND MA) independently masked for tumor type (primary or recurrent) and the mean scores used for analysis; the scores for both reviewers were nearly identical for all specimens and when there was discrepancy, consensus was reached. For S-100 staining, the percentage of positively staining cells within the enlarged plexiform nerves (excluding the surrounding matrix), and for diffuse neurofibroma the positive spindle cells in the tumor matrix were estimated. For KI-67, the percentage of positively staining cells at a fixed magnification (x20) was estimated. For Alcian blue, CD44, CD117, smooth muscle actin (SMA), and neurofilament labeling we classified the positively stained areas into five categories: Zero if there was no staining in the tissue section, as +1 if the cells or tissue in the tissue section showing staining less than 25%, as +2 if stain was between 25–50%, and if between 50–75% as +3, and +4 if more than 75%.

The data was collected on a pretested data collection form and transferred to a Microsoft Access spreadsheet. Univariate analysis was carried out using Statistical Package for Social Studies (SPSS 25) (IBM, Chicago, USA). For qualitative variables, we estimated frequencies and percentage proportions. To compare the outcomes in two subgroups we calculated Odds Ratio, its 95% confidence interval and two-sided P value. For more than two subgroups, we estimated chi-square value, degree of freedom and two-sided P value. For quantitative variables, we plotted a graph to study distribution. If the distribution was normal, we calculated the mean and standard deviation. To compare quantitative outcomes in subgroup, we estimated difference of mean its 95% confidence interval and two-sided P value. If the data was not distributed normally, we calculated the median and interquartile range. To validate the comparison of outcomes in related subgroups, we used Wilcoxon P value. A P value of ≤ 0.05 was considered as statistically significant.

For paired tumors, we used a 2 x 2 table to compare primary and recurrent tumors and their histologic subtype. Odds ratio, 95% confidence interval and two-sided P values were calculated. A P value of <0.05 was considered as statistically significant.

We also investigated the relationship between age and the primary histologic tumor type. The median age and its inter quartile range was estimated for plexiform and diffuse+ mixed type of tumors and compared using the nonparametric Mann Whitney U test for calculating the two-sided P value.

## Results

We identified 43 OPPN specimens from the KKESH ophthalmic pathology department archives obtained from 26 patients who underwent primary or recurrent tumor debulking/excision surgery from 2002 to 2018 at KKESH with a diagnosis of orbital-periorbital NF. Of the 43 specimens included in this study, 33 samples had formalin-fixed paraffin-embedded blocks available for further studies described below. Ten primary NF specimens had detailed pathology reports generated at KKESH by an experienced ophthalmic pathologist, but slides and tissue blocks were unavailable.

In summary, we studied the clinical and histopathological pattern of the 43 specimens (20 primary and 23 recurrent) from 26 patients. Of the 26 patients, we had 17 patients with paired tumors with both primary and recurrent tumor samples from the same patient. Three of the 26 patients only had a primary tumor excised with no recurrence and 6 patients in the recurrent group the primary tumor was excised elsewhere with no pathologic report of the primary tumor available; those 9 patients had tissue available that was used for histochemical and IHC studies. In cases where we had tissue specimens available (n = 33) we performed IHC stains. Alcian blue staining was performed for 14 specimens.

For the IHC studies, we had 10 primary and 23 recurrent tumors. The recurrent tumors were greater in number because tissues from primary tumors were unavailable because old paraffin blocks being disposed according to the hospital policy. Alcian blue stain was performed on 6 primary and 8 recurrent tumors.

The clinical presentation is summarized in (Table 1). The 26 patients included 13 males and 13 females (1:1 ratio). The median age at first presentation was 15 years, ranging from 2 to 53 years.

**Table 1:** Clinical presentation of 26 patients with orbital-periorbital plexiform neurofibromas.

		Count	%
Affected Side	Right	11	42.3
	Left	15	57.7
Number of Surgical Debulking	1	3	11.5
	2 to 3	15	57.7
	4 and more	8	30.8
Clinical Presentation	Lump/swelling	26	100
	Eyelid ptosis	23	88.5
	Eyebrow ptosis	1	3.8
	Proptosis	5	19.2
Location of Tumor	Upper eyelid	22	84.6
	Lower eyelid	4	15.4
	Eyebrow	6	23.1
	Orbit	5	19.2
The Extent of Surgical Resection	Total	10	38.5
	Subtotal	16	61.5

During surgery, 10 tumors were grossly resected totally (38.5%), and 16 tumors had subtotal resection (61.5%) as per surgical records. More than one surgical debulking was necessary for 23 patients (88.5%) and 8 of them (30.8%) needed more than 4 surgeries. The intervals between the primary and recurrent NF surgeries ranged between 3 months up to 12 years.

**Fig 1:**
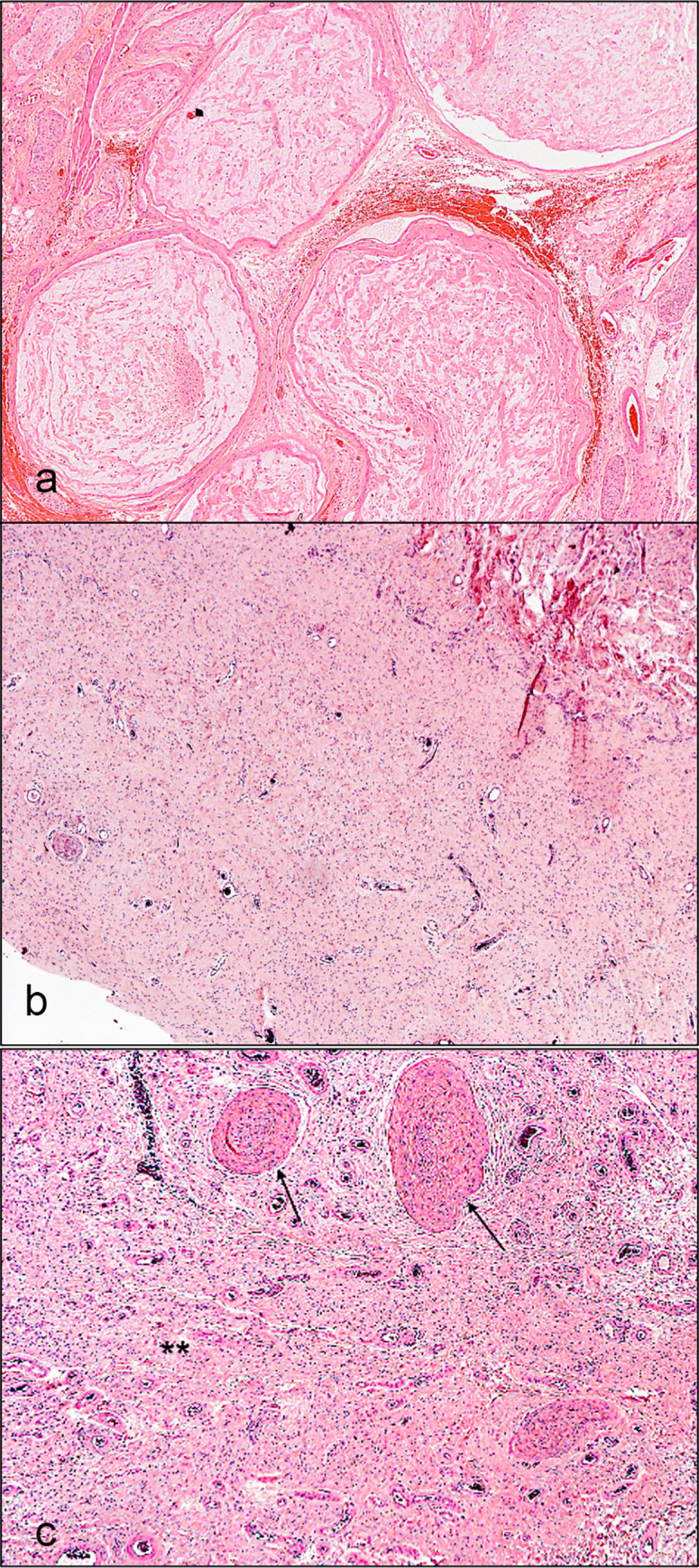
Microphotograph at low magnification showing different histologic types of primary periocular neurofibroma. **a** Primary plexiform neurofibroma showing discrete neurofibromas with Schwann cells and endoneurial fibroblasts within a myxoid matrix surrounded by perineurium (magnification x4). **b** Primary diffuse neurofibroma showing uniform extracellular matrix with collagen (magnification x4). **c** Primary mixed neurofibroma showing plexiform (arrows) and diffuse (asterisks) components (magnification x4). (all hematoxylin and eosin).

**Fig 2:**
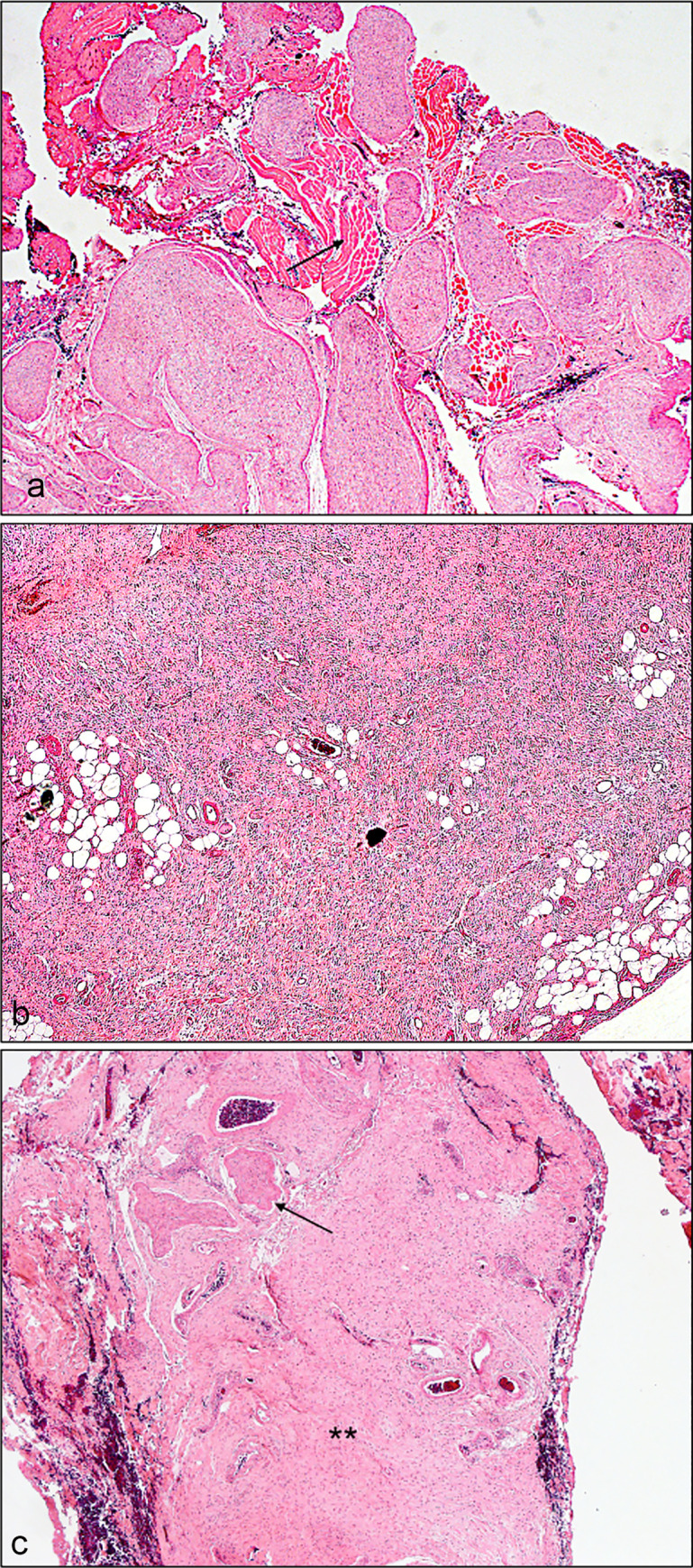
Microphotograph at low magnification showing different histologic types of recurrent neurofibroma. **a** Recurrent plexiform neurofibroma with infiltration of the periocular skeletal muscle (arrow) (magnification x2). **b** Recurrent diffuse neurofibroma (magnification x4). **c** Recurrent mixed neurofibroma showing plexiform (arrow) and diffuse (asterisks) components (magnification x4). (all hematoxylin and eosin).

On histopathological evaluation, plexiform NFs showed classic Schwann cells and fibroblasts in a myxoid matrix surrounded by perineurium. However, diffuse NFs showed infiltration of Schwann cells and fibroblasts to adjacent normal structures (muscles, glands, adipose tissue) in a compact matrix. Mixed NFs mostly exhibited diffuse NF features with focal areas of plexiform differentiation (Figs 1 and 2). Other cellular components were also seen within NFs such as axons, mast cells, and blood vessels.

For analysis, we combined the mixed and diffuse tumors (termed as diffuse in descriptions below) since the mixed tumors were mainly diffuse with focal areas that showed a plexiform pattern. Of the primary NFs (n = 20) there were 13 plexiform (65%), and 7 diffuse (35%) NFs. Of the recurrent NFs (n = 23), there were 5 plexiform (21.7%), and 18 diffuse (78.3%) NFs (Table 2).

**Table 2:** Distribution of 43 neurofibroma specimens in 26 patients with primary and recurrent tumors.

	Primary (n = 20; 46.5%)		Recurrent (n = 23; 53.5%)		Total (n = 43; 100%)	
*P = 0*.*005*						
	Count	%	Count	%	Count	%
Plexiform	13	65	5	21.7	18	41.9
Diffuse	6	30	13	56.5	19	44.2
Mixed	1	5	5	21.7	6	13.9

Of the 17 patients with paired tumors, 12/17 (70.6%) primary NFs were plexiform and 5/17 (29.4%) were diffuse, however when they recurred in the 17 cases, 13/17 tumors (76.5%) showed a diffuse pattern and only 4/17 tumors (23.5%) had a plexiform pattern suggesting a reversal in the dominant histologic pattern from plexiform to a diffuse pattern (Table 3). The ratio of primary NFs by histopathological pattern was 13:7 plexiform: diffuse whereas the ratio of plexiform NFs to diffuse NFs in recurrent tumors 3:8 after the first recurrence, and after multiple recurrences was 1:5 suggesting that there was an increased likelihood of seeing a diffuse pattern after additional tumor excision/debulking. For the paired samples, the odds of recurrent tumors being diffuse in pattern was significantly higher than having a plexiform pattern [Odds ratio (OR) = 7.8 (95% confidence interval 1.69: 36.1) *P = 0*.*008*].

**Table 3:** Distribution of 34 neurofibromas in 17 patients with paired primary and recurrent tumors.

	Primary (n = 17)		Recurrent (n = 17)	
	Count	%	Count	%
Plexiform	12	70.6	4	23.5
Diffuse	4	23.5	9	52.9
Mixed	1	5.9	4	23.5

The median age of 13 primary NFs with plexiform type was 6 years (IQR 3.5; 16). The median age of 7 primary NFs with diffuse type was 18 years (IQR 15; 24). The age at initial surgery was significantly different between the two histologic subtypes (Mann Whitney U P = 0.014).

**Fig 3:**
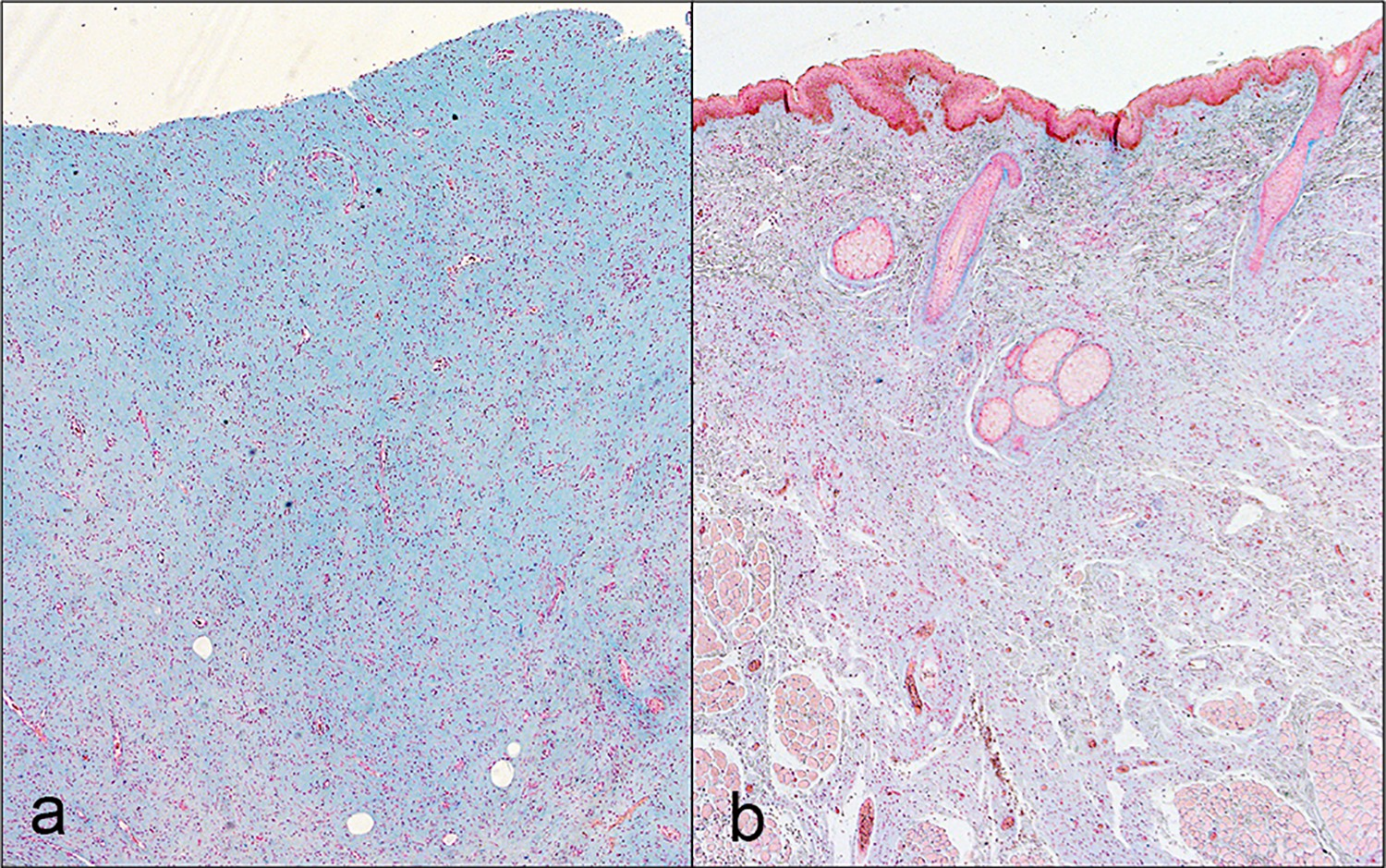
Representative Alcian blue staining patterns showing diffuse staining of greater intensity in the matrix of primary diffuse neurofibromas compared to recurrent tumors. **a** Primary diffuse neurofibroma (magnification x4). **b** Recurrent diffuse neurofibroma showing diffuse staining of lower intensity (magnification x4).

Examination of Alcian blue staining in 14 representative cases showed a significantly higher intensity of Alcian blue staining in the extracellular matrix of primary (plexiform and diffuse) NFs when compared to the recurrent group (*P = 0*.*03*) (Fig 3). When we compared the staining intensity between plexiform and diffuse NFs in primary and recurrent cases, it appeared higher in intensity in the plexiform group but was not statistically significant (*P = 0*.*42*) (Table 4).

**Table 4:** Immunohistochemical and histochemical staining in different types of neurofibromas.

	Primary	Recurrent	P values	Plexiform	Diffuse	P values
**S-100** ^**a**^	100	86.1 ± 17.7	*P = 0*.*02*	93.9 ±16.6	88 ± 16	*P = 0*.*6*
**CD117** ^**b**^	1.1 ± 0.9	2.2 ± 1.1	*P = 0*.*01*	1.1 ± 1.0	2.4 ± 1.0	*P<0*.*001*
**CD44** ^**b**^	0.6 ± 0.8	1.3 ± 0.7	*P = 0*.*02*	0.7 ± 0.8	1.38 ± 0.7	*P = 0*.*08*
**Smooth muscle actin** ^**b**^	0.5 ± 0.7	0.5 ± 0.6	*P = 0*.*86*	0.9 ± 0.7	0.3 ± 0.4	*P = 0*.*02*
**Neurofilament** ^**b**^	1.4 ± 1.0	2.8 ± 0.8	*P<0*.*001*	2.1 ± 1.2	2.5 ± 1.0	*P = 0*.*55*
**Ki-67** ^**a**^	0.25 ± 0.4	0.37 ± 0.3	*P = 0*.*3*	0.27 ± 0.3	0.4 ± 0.3	*P = 0*.*35*
**Alcian blue** ^**b**^	3.0 ± 1.3	1.4 ± 1.1	*P = 0*.*03*	2.7 ± 1.2	1.63 ± 1.5	*P = 0*.*42*

^a^ Results are percentage (0–100%).

^b^ Results are classified as: 0: No staining +1: <25% +2: 25–50% +3: 50–75% +4: >75%.

**Fig 4:**
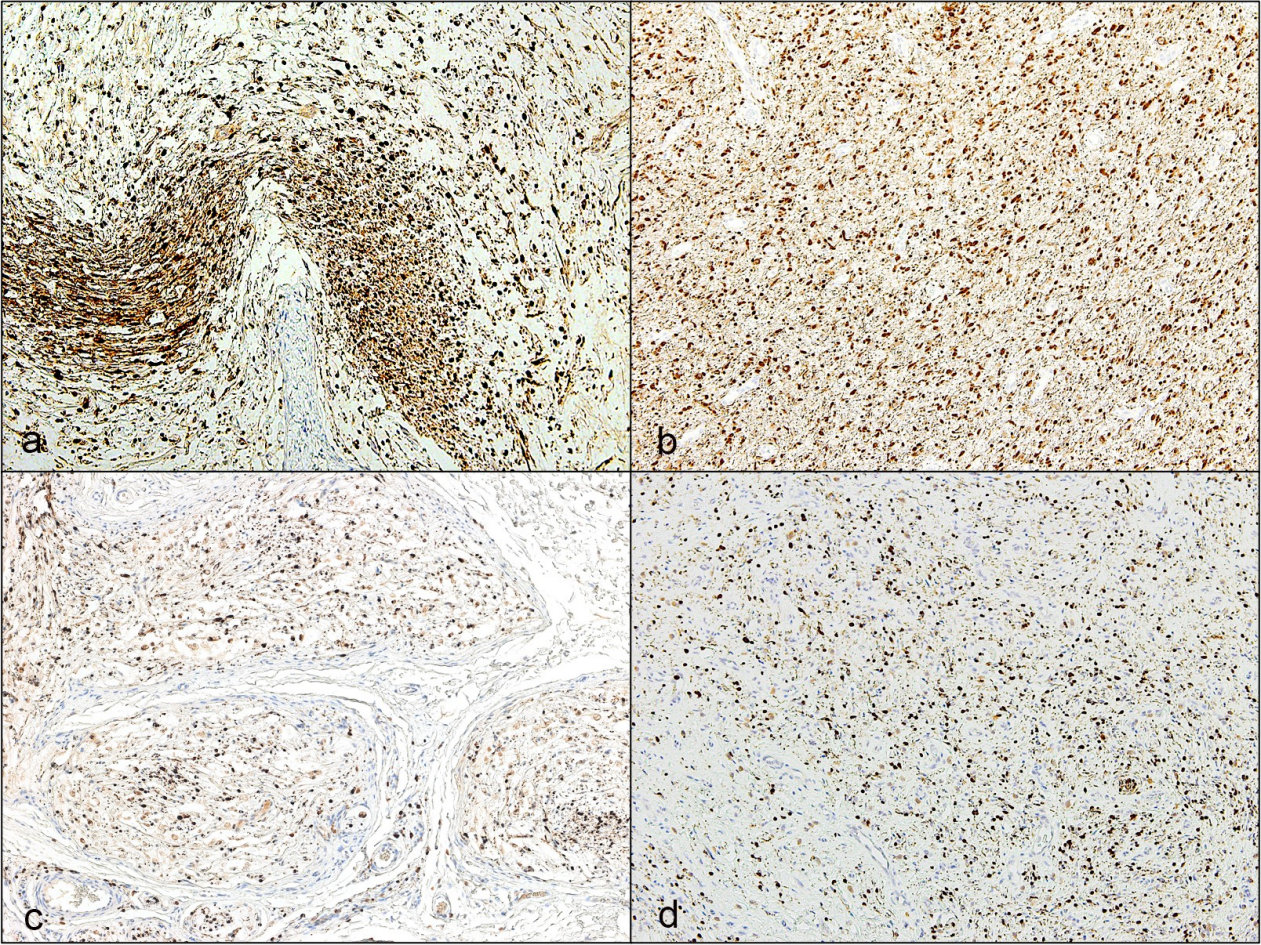
Representative immunostaining patterns for S-100 positive Schwann cells in primary and recurrent neurofibromas. **a** Primary plexiform neurofibroma showing large areas of S-100 positive spindle cells (magnification x10). **b** Primary diffuse neurofibroma showing large areas of S-100 positive spindle cells (magnification x10). **c** Recurrent plexiform neurofibroma showing decrease in S-100 positive spindle cells (magnification x10). **d** Recurrent diffuse neurofibroma showing decrease in S-100 positive spindle cells (magnification x10). (chromogen diaminobenzidine).

IHC staining with S-100 protein antibody, which labels Schwann cells, showed strong immunoreactivity of spindle cells in all specimens with variable areas of staining (Fig 4). Primary (plexiform and diffuse) NFs showed significantly higher areas of spindle cell labeling with S-100 protein compared to recurrent tumors (100% vs 86%) *(P = 0*.*02*). However, areas of S-100 labeling in plexiform and diffuse (primary or recurrent) NFs were not significantly different (*P = 0*.*6)* (Table 4).

**Fig 5:**
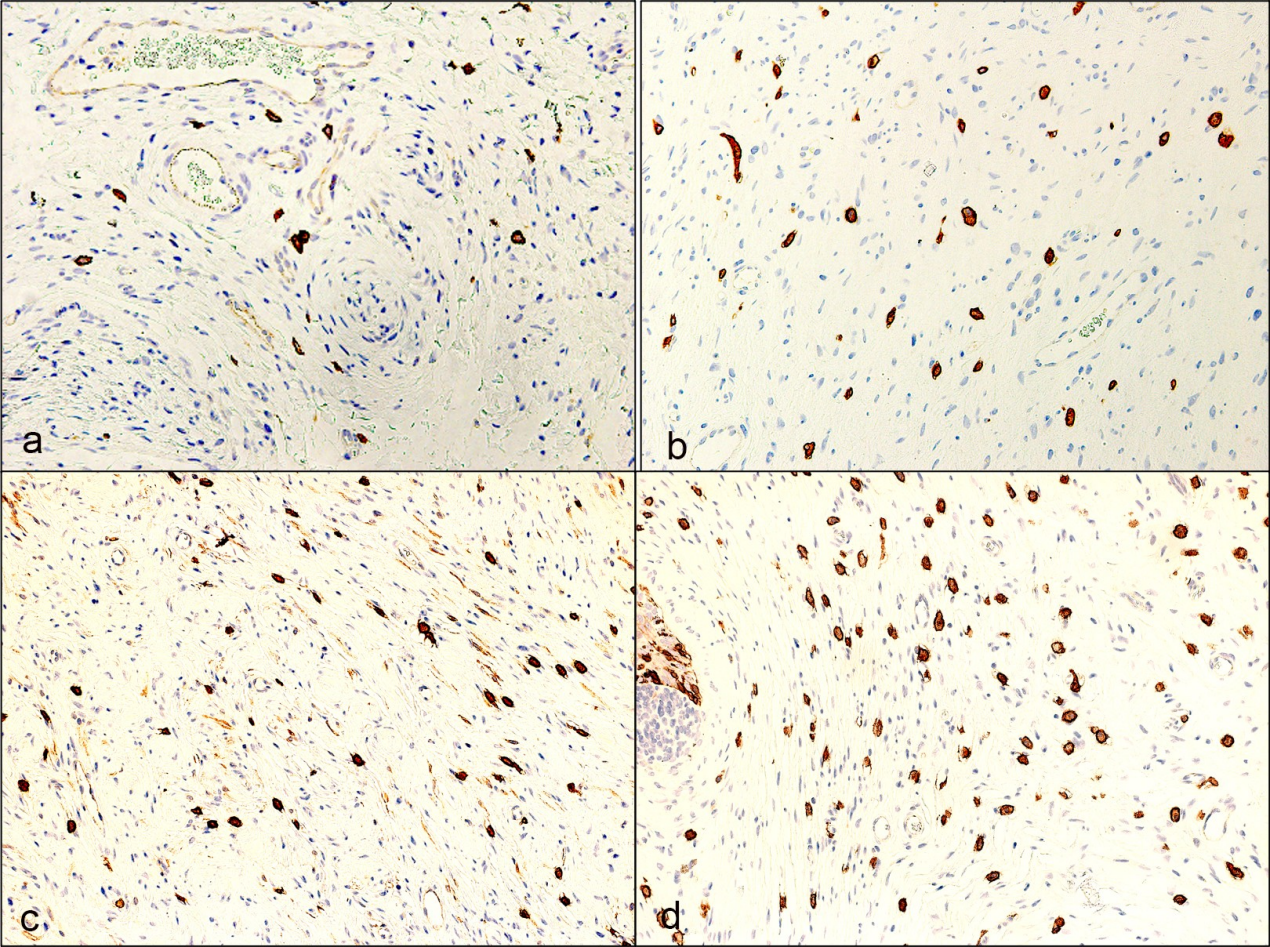
Representative immunostaining patterns for CD117 positive mast cells in primary and recurrent neurofibromas. **a** Primary plexiform neurofibroma showing grade +1 around a plexiform neurofibroma (magnification x20). **b** Primary diffuse neurofibroma showing grade +1.5 (magnification x20). **c** Recurrent diffuse neurofibroma showing grade +2 in the second recurrence (magnification x20). **d** Recurrent diffuse neurofibroma showing grade +3 in the fourth recurrence (magnification x20). (chromogen diaminobenzidine).

CD117, a mast cell marker that plays a role in NFs [22,23]. Larger areas of CD117 cellular labeling were noted in recurrent (plexiform and diffuse) NFs compared to primary tumors (*P = 0*.*01*). The labeling appeared to qualitatively increase with each recurrence (Fig 5). Diffuse NFs in primary and recurrent cases had significantly larger areas of CD117 labeling than plexiform NFs (*P<0*.*001)* (Table 4). When the recurrence was a transformation from a plexiform to mixed type NF, CD117 was almost absent in the primary plexiform areas and prominent in the areas of diffuse NF in the recurrent tumor.

**Fig 6:**
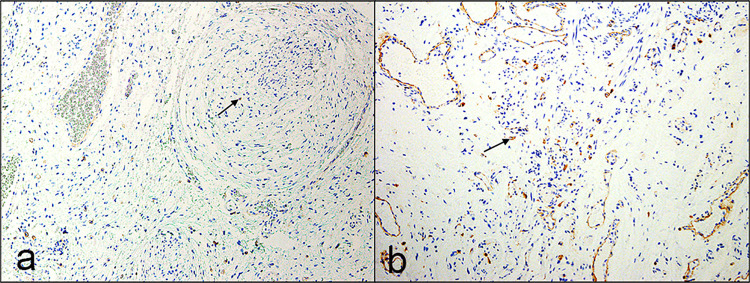
Representative immunostaining patterns for CD44 positive cells in primary and recurrent neurofibromas. Note the cytoplasmic membrane staining typical of CD44 and spindle cells staining. **a** Primary plexiform neurofibroma showing scarce CD44 positive cells (grade +0.5) (magnification x10). **b** Recurrent diffuse neurofibroma showing grade +2 positive cells (magnification x10). (chromogen diaminobenzidine).

CD44 immuno-reactivity was variable in NFs from being absent to reaching up to 75% of the cell population with no significant difference among NF patterns (*P = 0*.*08*) (Fig 6). Recurrent (plexiform and diffuse) NFs had significantly higher CD44 areas of labeling than primary tumors (*P = 0*.*02*) (Table 4).

**Fig 7:**
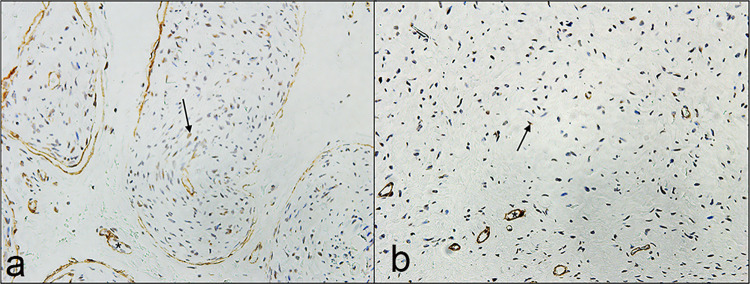
Representative immunostaining patterns for smooth muscle actin positive myofibroblast cells in primary and recurrent neurofibromas. Note typical staining in pericytes around blood vessels (asterisk) with few spindle cells staining (arrow). **a** Primary plexiform neurofibroma showing grade +2 staining in spindle cells (magnification x20). **b** Recurrent diffuse neurofibroma showing grade +0.5 staining in spindle cells (magnification x20). (chromogen diaminobenzidine).

Smooth muscle actin (SMA), a myofibroblast marker [24], showed absent immunoreactivity in 42% of NFs and low immunopositivity in the rest of the tumors (Fig 7). There was no significant difference between primary and recurrent (plexiform and diffuse) NFs (*P = 0*.*86*). However, plexiform NFs (primary and recurrent, combined) were found to have significantly greater areas of labeling than diffuse tumors (primary and recurrent, combined) (*P = 0*.*02)* (Table 4).

**Fig 8:**
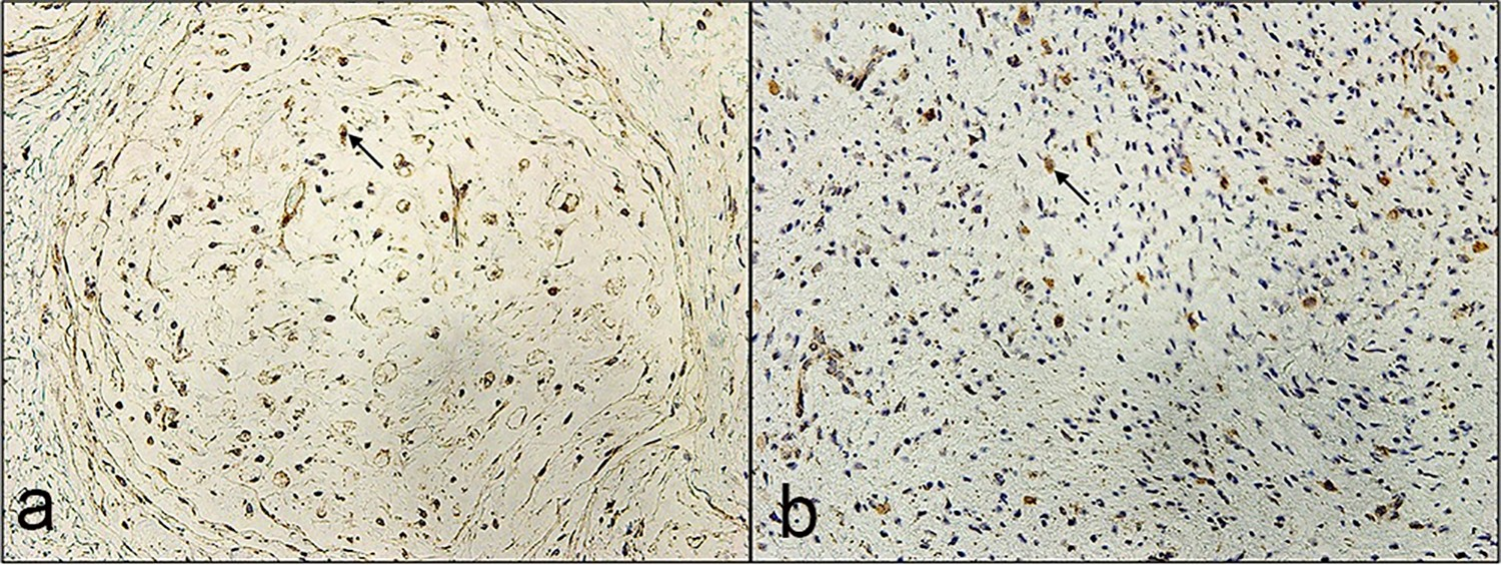
Representative immunostaining patterns for neurofilament protein positive cells in primary and recurrent neurofibromas (arrow). **a** Primary plexiform neurofibroma showing grade +1 (magnification x20). **b** Recurrent diffuse neurofibroma showing grade +2 (magnification x20). (chromogen diaminobenzidine).

Neurofilament protein, an axon marker, showed variable expression between the tumors with significantly larger areas of axonal labeling in recurrent (plexiform and diffuse) NFs compared to primary tumors (*P<0*.*001*) (Fig 8). Also, the primary plexiform tumors showed labeling that was confined to axons in the plexiform component of the tumor whereas in primary and recurrent diffuse the NF labeled axons were scattered in the cellular matrix. Though we did not quantify the number of axons labeled in each type it was felt that the larger area of staining in recurrent tumors that were diffuse in nature were as result of change in distribution rather than the increase in the number of axons. However, there was no significant difference in labeling between plexiform and diffuse NFs (primary and recurrent, combined) (*P = 0*.*55*) (Table 4).

Ki-67 proliferation index (PI) showed either no mitotic activity in 40% or 1% or less mitotic activity in 60% of NFs. PI was not significantly different between primary and recurrent NFs (*P = 0*.*3*) or between histologic types (*P = 0*.*35*) (Table 4).

## Discussion

In this study we show that the primary tumors of 17 paired OPPNs were typically plexiform NFs (70.6%) and were more common than the diffuse type. However, after surgical debulking in recurrent tumors, 66.7% of plexiform NFs lost the pattern seen in primary tumors with transformation to a diffuse pattern during recurrence and became the most common pattern in most of the cases (76.5%). Furthermore, it appeared that the likelihood of the change from plexiform to diffuse pattern increased with the number of debulking procedures. Friedrich et al. [25] studied 25 NFs in head and neck, breast, back, and extremities of NF1 patients and the NF type did not differ between primary lesions and recurrent tumors. Unfortunately, in this study no data was provided on how many tumors were OPPN and therefore direct comparisons between our study and this report was not possible.

S-100 protein-labeled Schwann cells are reported to be variably immunoreactive in all types of NFs [13,19,26–28]. In this study, all cases of primary (plexiform and diffuse) NFs demonstrated extensive Schwann cells immunopositivity in the spindle cells within the plexiform enlarged axons of plexiform tumors and the spindle cells within the stroma in diffuse tumors (100%), but recurrent tumors showed spindle cells that stained only 86% of the cell population in both plexiform and diffuse tumors. This decrease in area of staining in recurrent NFs was likely due to fewer spindle cells showing S-100 labeling and not a decrease in the number of spindle cells. There was also a shift in the pattern of spindle cell distribution from a more loosely arranged cells to a more compact pattern in diffuse tumors which may have influenced the density of S-100 labeling but not area of S-100 labeling in diffuse tumors which appeared more cellular.

The possible mechanisms involved in this unusual switch in histological pattern in recurrent OPPN was then investigated using an array of IHC stains to probe the cellular elements and extracellular matrix in primary and recurrent tumors.

Review and analysis of the clinical data revealed that the age of excision of patients with primary diffuse tumors was significantly higher when compared to primary plexiform tumors. The reasons for the age differences at the time of surgical intervention in this small cohort with an unequal sample size of plexiform and diffuse primary tumors, is not entirely clear. One could possibly hypothesize that diffuse NF tumors were slow growing and not that disfiguring as the plexiform variant, and hence the later age of excision for diffuse tumors. Whether age played an independent role in the structural differences between the primary and recurrent tumors is difficult to dissect in this small cohort.

The reduction in S-100 labeled spindle cells in recurrent tumors could have several explanations. Based on the IHC studies, the most likely explanation is that these S-100 negative spindle cells represent proliferation of NF1 related fibroblasts stimulated by mast cells. NFs contain three major types of neural tissue derived cells: Schwann cells, fibroblasts and perineurial cells [29]. The presence and role of mast cells in NFs has been well described [22,23]. It has been suggested that mast cells secrete transforming growth factor β (TGF-β) which in turn causes fibroblast migration proliferation and collagen synthesis by these fibroblasts [30]. Though we did not specifically label the spindle cells for fibroblast markers, we noted that these spindle cells were not myofibroblastic in nature since the areas of smooth muscle actin expression [24] in spindle cells did not increase in recurrent tumors. On the other hand, there was a significant increase in number of mast cells in recurrent tumors that appeared to increase with each recurrence and suggests these cells may play a role in the proliferation of spindle cell fibroblasts and the conversion to a diffuse pattern represented by fibroblasts, and not spindle cells. Increase in mast cells density been described in diffuse NFs [22]. Another contribution to the S-100 negative spindle cell population could have been EMA positive perineurial cells [31] but this was not investigated in this study.

An additional explanation for a decrease in S-100 labeled Schwann cells could be related to the injury to Schwann cells caused by surgical excision. Schwann cells lose S-100 staining in peripheral nerve injuries when mature Schwann that label with S-100, dedifferentiate back to an immature state and lose S-100 immunoreactivity [32,33]. This change described in the report is likely an acute event and is unlikely to be an explanation for loss of spindle cell S-100 standing in our study.

We also investigated the proliferative nature of the cellular components using Ki-67 labeling which was low [19,25,27,34] and comparable in primary and recurrent tumors reflecting the clinical behavior of recurrent tumors which is slow and not rapid growth in recurrent NFs. Friedrich et al. [25] showed that using Ki-67 staining, the proliferative index of most NF specimens were less than 1% with no association between primary and recurrent tumors or between the different tumor patterns, similar to that seen in our series. Furthermore, a case report also compared a primary with recurrent plexiform NF found that there was no change in tumor pattern and that proliferative index was 0% in the primary tumor but reaching 5% in the recurrence [35].

The extracellular myxoid matrix in plexiform NF’s is usually loose, whereas in diffuse NFs it is more compact and the loose myxoid matrix labels with Alcian blue [8]. Our study showed significantly less intense Alcian blue staining in the recurrent NFs compared to the primary (*P = 0*.*03*). This observation was not surprising since most recurrent NFs were diffuse in nature with paucity of loose mucoid extracellular matrix and abundance of a compact collagenous matrix.

CD44, a transmembrane glycoprotein receptor for hyaluronic acid participates in cell-extracellular matrix interactions and migration [36–40]. CD44 may play a vital role in NF1 [36] related to the invasion and promotion of metastases of tumors with malignant transformation, through the alteration of their ability to infiltrate adjacent tissue. Riddle et al. [36] found that malignant peripheral nerve sheath tumors (MPNSTs) were strongly positive for CD44; however, most of the NFs demonstrated only focal immunopositivity, which was most intense in infiltrative and non-encapsulated tumors. In our series, CD44 labeling was significantly higher in the spindle cells of recurrent tumors (*P = 0*.*02*). Though these recurrent tumors in our study were not malignant, the increase in CD44 expression might reflect the infiltrative nature of recurrent tumors that are mainly diffuse in nature.

Neurofilament protein is a neural marker targeting nerve axons and dendrites typically found in neural tumors like NF [13,41–43]. Wechsler et al. [44] found that all NFs were immunopositive for neurofilament protein however, Ghilusi et al. [43] found that only 40% were immunopositive. In our study all tumors showed neurofilament protein labeling with significantly higher expression in recurrent NFs compared to the primary tumors (*P<0*.*001*). The increase in neurofilament protein in recurrent tumors could represent new axons sprouts following surgical injury during excision.

Clinically, the most common clinical presentation of OPPN was a lump/swelling in all cases, ptosis of the upper eyelid in 88.5%, followed by proptosis in 19.2%. These findings were similar to those reported in the literature [4,5]. There was no sex preference, and the tumor was almost always isolated to one side of the face and recurrences did not show a specific clinical phenotype. Only a few cases have been reported showing a bilateral OPPN [4,12,45,46]. Surgical resection remained the mainstay of treatment for OPPN [5]; however, surgeons find it difficult to achieve a complete tumor excision without complications due to the infiltrative nature of the tumor and ability to define margins. About 60% of tumors had subtotal resection either due to extensive and infiltrative growth behavior of the tumor or the involvement of vital structures, e.g. nerves or muscles and that was similar to that reported in the literature [15]. Our observation that recurrent tumors are more likely to be diffuse suggests that removal of such tumors in total when they recur may be challenging and is reflected in the observation that 88.5% of patients needed multiple debulking procedures in our series. Needle et al. [15], in a 20 year retrospective study reported that of the 168 NFs in pediatric patients, 43% of tumors recurred after surgery and that higher recurrence was associated with children less than 10 years old (60%), subtotal resection (45%) and tumors of head, neck, and face. The need for repeated surgical debulking of NFs in the orbital-periorbital region was 88.5% in our series which is higher than what was previously published in the literature (15–23 and 43%) [15–17] but these recurrences reported were not in the orbital-periorbital region and hence likely the reason for the lower recurrence rate.

One limitation of this study is a small number of patients that were studied retrospectively. Also, the lack of a robust number of paired samples for analysis is a minor limitation; however, the conversion rate from plexiform to diffuse tumors appeared in the paired samples was evident. Whether a similar conversion of histologic type happens in recurrent NFs elsewhere in the body, remains to be studied.

There seems to be a high risk for primary periocular plexiform NFs to transform into a diffuse pattern when they recur. This observation has not been previously reported. We suggest that following surgical injury, mast cells may play a potential role in that transformation process leading to non-Schwann cell spindle proliferation, a compact collagenous matrix and a CD44 related ability of the tumor to become infiltrative in nature. Further laboratory-based experiments are needed to shed further light into the specific mechanisms involved in this morphologic switch.

**Appendix 1:** Immunohistochemical markers.

Antibody	Company (Cat#)	Dilution	Role
S-100	SensiStainTM (HY000226)	1:500	Identifying Schwann cells; the main component of neurofibroma [13].
CD117	Abcam (ab32363)	1:100	Identifying mast cells within the tumor [22,23].
CD44	SensiStainTM (HY000056)	1:100	Assessing growth and infiltrative pattern of the tumor [36–40].
Smooth muscle actin	GenomeMe (IHC506-100)	1:200	Marker for myofibroblasts which have an important role in wound healing, fibrosis, and scar formation [24].
Neurofilament	Abcam (ab7795)	1:100	Delineate small axons within the tumor [13,41].
Ki-67	SensiStainTM (HY000157)	1:100	Proliferation index (PI) measuring the mitotic activity of the tumor [25,35].

## Supporting information

S1 File (XLSX)

## References

[1] Riccardi V, Smirniotopoulos J. Neurofibromatosis, phenotype, natural history, and pathogenesis. Journal of Neuropathology and Experimental Neurology. 1992;51(6).

[2] Holtkamp N, Mautner V-F, Friedrich RE, Harder A, Hartmann C, Theallier-Janko A, et al. Differentially expressed genes in neurofibromatosis 1-associated neurofibromas and malignant peripheral nerve sheath tumors. Acta neuropathologica. 2004;107(2):159–68. doi: 10.1007/s00401-003-0797-8 14673600

[3] Cavenee WK, Louis DN, Ohgaki H, Wiestler OD. WHO classification of tumours of the central nervous system: WHO Regional Office Europe; 2007. 10.1007/s00401-007-0243-4 PMC1929165 17618441

[4] Chaudhry I, Morales J, Shamsi F, Al-Rashed W, Elzaridi E, Arat Y, et al. Orbitofacial neurofibromatosis: clinical characteristics and treatment outcome. Eye. 2012;26(4):583. doi: 10.1038/eye.2011.336 22193879 PMC3325560

[5] Avery RA, Katowitz JA, Fisher MJ, Heidary G, Dombi E, Packer RJ, et al. Orbital/periorbital plexiform neurofibromas in children with neurofibromatosis type 1: multidisciplinary recommendations for care. Ophthalmology. 2017;124(1):123–32. doi: 10.1016/j.ophtha.2016.09.020 27817916 PMC5173440

[6] Scheithauer BW, Woodruff JM, Erlandson RA. Tumors of the peripheral nervous system: Amer Registry of Pathology; 1999.

[7] Korf BR. Plexiform neurofibromas. American journal of medical genetics. 1999;89(1):31–7. doi: 10.1002/(sici)1096-8628(19990326)89:1&lt;31::aid-ajmg7&gt;3.0.co;2-w 10469434

[8] Goldblum J, Folpe A. Weiss ShW. Enzinger and Weiss’s Soft tissue tumors 6-th edition. Philadelphia: Saunders/Elsevier; 2014.

[9] Shields JA, Shields CL. Eyelid, conjunctival, and orbital tumors: An atlas and textbook: Lippincott Williams & Wilkins; 2008.

[10] Garrity JA, Henderson JW, Cameron JD. Henderson’s orbital tumors: Lippincott Williams & Wilkins; 2007.

[11] Alkatan HM. Solitary neurofibroma in the absence of neurofiromatosis. Canadian Journal of Ophthalmology. 2007;42(4):628–9. doi: 10.3129/canjophthalmol.i07-087 17641714

[12] Alshomar KM, Alkatan HM, Alsuhaibani AH. Bilateral orbital isolated (solitary) neurofibroma in the absence of neurofibromatosis–A case report. Saudi Journal of Ophthalmology. 2018;32(1):83–5. doi: 10.1016/j.sjopt.2018.02.002 29755279 PMC5944016

[13] Friedrich RE, Holstein A-F, Middendorff R, Davidoff MS. Vascular wall cells contribute to tumourigenesis in cutaneous neurofibromas of patients with neurofibromatosis type 1. A comparative histological, ultrastructural and immunohistochemical study. Anticancer research. 2012;32(5):2139–58. 22593502

[14] Arnold A, Imada EL, Zhang L, Edward DP, Marchionni L, Rodriguez FJ. Differential gene methylation and expression of HOX transcription factor family in orbitofacial neurofibroma. Acta neuropathologica communications. 2020;8:1–11. doi: 10.1186/s40478-019-0875-3 32366326 PMC7197183

[15] Needle MN, Cnaan A, Dattilo J, Chatten J, Phillips PC, Shochat S, et al. Prognostic signs in the surgical management of plexiform neurofibroma: the Children’s Hospital of Philadelphia experience, 1974–1994. The Journal of pediatrics. 1997;131(5):678–82. doi: 10.1016/s0022-3476(97)70092-1 9403645

[16] McCarron KF, Goldblum JR. Plexiform neurofibroma with and without associated malignant peripheral nerve sheath tumor: a clinicopathologic and immunohistochemical analysis of 54 cases. Modern pathology: an official journal of the United States and Canadian Academy of Pathology, Inc. 1998;11(7):612–7. 9688181

[17] Nguyen R, Ibrahim C, Friedrich RE, Westphal M, Schuhmann M, Mautner V-F. Growth behavior of plexiform neurofibromas after surgery. Genetics in Medicine. 2013;15(9):691. doi: 10.1038/gim.2013.30 23598713

[18] Health NIo. Consensus Development Conference Statement: neurofibromatosis. Bethesda, Md., USA, July 13–15, 1987. Neurofibromatosis. 1988;1(3):172–8. 3152465

[19] Zhang ML, Suarez MJ, Bosley TM, Rodriguez FJ. Clinicopathological features of peripheral nerve sheath tumors involving the eye and ocular adnexa. Human pathology. 2017;63:70–8. doi: 10.1016/j.humpath.2017.02.006 28235631 PMC5517313

[20] Staser K, Yang F-C, Clapp DW. Pathogenesis of plexiform neurofibroma: tumor-stromal/hematopoietic interactions in tumor progression. Annual Review of Pathology: Mechanisms of Disease. 2012;7:469–95. doi: 10.1146/annurev-pathol-011811-132441 22077553 PMC3694738

[21] Babovic-Vuksanovic D, Messiaen L, Nagel C, Brems H, Scheithauer B, Denayer E, et al. Multiple orbital neurofibromas, painful peripheral nerve tumors, distinctive face and marfanoid habitus: a new syndrome. European Journal of Human Genetics. 2012;20(6):618. doi: 10.1038/ejhg.2011.275 22258529 PMC3355267

[22] Tucker T, Riccardi VM, Sutcliffe M, Vielkind J, Wechsler J, Wolkenstein P, et al. Different patterns of mast cells distinguish diffuse from encapsulated neurofibromas in patients with neurofibromatosis 1. Journal of Histochemistry & Cytochemistry. 2011;59(6):584–90. doi: 10.1369/0022155411407340 21525187 PMC3201189

[23] Yang F-C, Staser K, Clapp DW. The plexiform neurofibroma microenvironment. Cancer Microenvironment. 2012;5(3):307–10. doi: 10.1007/s12307-012-0115-x 22821631 PMC3460056

[24] Mahale A, Fikri F, Al Hati K, Al Shahwan S, Al Jadaan I, Al Katan H, et al. Histopathologic and immunohistochemical features of capsular tissue around failed Ahmed glaucoma valves. PloS one. 2017;12(11):e0187506. doi: 10.1371/journal.pone.0187506 29121102 PMC5679546

[25] Friedrich R, Hagel C, Brehme Z, Kluwe L, Mautner V. Ki-67 proliferation-index (MIB-1) of neurofibromas in neurofibromatosis type 1 patients. Anticancer research. 2003;23(2A):953–5. 12820329

[26] Blessmann M, Gröbe A, Quaas A, Kaifi JT, Mistakidis G, Bernreuther C, et al. Adhesion molecule L1 is down-regulated in malignant peripheral nerve sheath tumors versus benign neurofibromatosis type 1–associated tumors. Oral surgery, oral medicine, oral pathology and oral radiology. 2012;113(2):239–44. doi: 10.1016/j.tripleo.2011.04.019 22677742

[27] Luscan A, Masliah-Planchon J, Laurendeau I, Ortonne N, Varin J, Lallemand F, et al. The activation of the WNT signaling pathway is a Hallmark in neurofibromatosis type 1 tumorigenesis. Clinical Cancer Research. 2014;20(2):358–71. doi: 10.1158/1078-0432.CCR-13-0780 24218515

[28] Stagner AM, Jakobiec FA. Peripheral nerve sheath tumors of the eyelid dermis: a clinicopathologic and immunohistochemical analysis. Ophthalmic plastic and reconstructive surgery. 2016;32(1):40–5. doi: 10.1097/IOP.0000000000000424 25794028

[29] Peltonen J, Jaakkola S, Lebwohl M, Renvall S, Risteli L. Cellular differentiation and expression of matrix genes in type 1 neurofibromatosis. Lab Invest. 1988;1998159. 2462129

[30] Staser K, Yang F-C, Clapp DW. Mast cells and the neurofibroma microenvironment. Blood. 2010;116(2):157–64. doi: 10.1182/blood-2009-09-242875 20233971 PMC2910605

[31] Hornick JL, Bundock EA, Fletcher CD. Hybrid schwannoma/perineurioma: clinicopathologic analysis of 42 distinctive benign nerve sheath tumors. The American journal of surgical pathology. 2009;33(10):1554–61. doi: 10.1097/PAS.0b013e3181accc6c 19623031

[32] Jessen KR, Mirsky R. Negative regulation of myelination: relevance for development, injury, and demyelinating disease. Glia. 2008;56(14):1552–65. doi: 10.1002/glia.20761 18803323

[33] Zhang S-j, Wu W-l, Yang K-y, Chen Y-z, Liu H-c. Phenotypic changes of Schwann cells on the proximal stump of injured peripheral nerve during repair using small gap conduit tube. Neural regeneration research. 2017;12(9):1538. doi: 10.4103/1673-5374.215266 29090001 PMC5649476

[34] Zhu Y, Ghosh P, Charnay P, Burns DK, Parada LF. Neurofibromas in NF1: Schwann cell origin and role of tumor environment. Science. 2002;296(5569):920–2. doi: 10.1126/science.1068452 11988578 PMC3024710

[35] Fujiwara M, Isaka F, Honjo G, Ozawa T, Tachibana T. Solitary plexiform neurofibroma: recurrence 32 years after excision. Journal of the European Academy of Dermatology and Venereology. 2006;20(6):756–7. doi: 10.1111/j.1468-3083.2006.01534.x 16836521

[36] Riddle ND, Gorden L, Rojiani MV, Hakam A, Rojiani AM. CD44 and p53 immunoexpression patterns in NF1 neoplasms-indicators of malignancy and infiltration. International journal of clinical and experimental pathology. 2010;3(5):515. 20606732 PMC2897111

[37] Su W, Gutmann DH, Perry A, Abounader R, Laterra J, Sherman LS. CD44‐independent hepatocyte growth factor/c‐Met autocrine loop promotes malignant peripheral nerve sheath tumor cell invasion in vitro. Glia. 2004;45(3):297–306. doi: 10.1002/glia.10340 14730703

[38] Wiranowska M, Ladd S, Smith SR, Gottschall PE. CD44 adhesion molecule and neuro-glial proteoglycan NG2 as invasive markers of glioma. Brain cell biology. 2006;35(2–3):159–72. doi: 10.1007/s11068-007-9009-0 17957481

[39] Su W, Sin M, Darrow A, Sherman LS. Malignant peripheral nerve sheath tumor cell invasion is facilitated by Src and aberrant CD44 expression. Glia. 2003;42(4):350–8. doi: 10.1002/glia.10206 12730955

[40] Ponta H, Sherman L, Herrlich PA. CD44: from adhesion molecules to signalling regulators. Nature reviews Molecular cell biology. 2003;4(1):33. doi: 10.1038/nrm1004 12511867

[41] Kawahara E, Oda Y, Ooi A, Katsuda S, Nakanishi I, Umeda S. Expression of glial fibrillary acidic protein (GFAP) in peripheral nerve sheath tumors. A comparative study of immunoreactivity of GFAP, vimentin, S-100 protein, and neurofilament in 38 schwannomas and 18 neurofibromas. The American journal of surgical pathology. 1988;12(2):115–20. doi: 10.1097/00000478-198802000-00004 3124642

[42] Eagle RC Jr. Immunohistochemistry in diagnostic ophthalmic pathology: a review. Clinical & Experimental Ophthalmology. 2008;36(7):675–88. 10.1111/j.1442-9071.2008.01870.x 18983554

[43] Ghilusi M, Plesea I, Comanescu M, Enache S, Bogdan F. Preliminary study regarding the utility of certain immunohistochemical markers in diagnosing neurofibromas and schwannomas. Rom J Morphol Embryol. 2009;50(2):195–202. 19434310

[44] Wechsler J, Lantieri L, Zeller J, Voisin M-C, Martin-Garcia N, Wolkenstein P. Aberrant axon neurofilaments in schwannomas associated with phacomatoses. Virchows Archiv. 2003;443(6):768–73. doi: 10.1007/s00428-003-0895-y 14508685

[45] Morales J, Chaudhry IA, Bosley TM. Glaucoma and globe enlargement associated with neurofibromatosis type 1. Ophthalmology. 2009;116(9):1725–30. doi: 10.1016/j.ophtha.2009.06.019 19729098

[46] Altan-Yaycioglu R, Hintschich C. Clinical features and surgical management of orbitotemporal neurofibromatosis: a retrospective interventional case series. Orbit. 2010;29(5):232–8. doi: 10.3109/01676831003660689 20812827

